# Identification and validation of prognostic genes and prognostic models associated with cutaneous melanoma and integrative stress response

**DOI:** 10.3389/fimmu.2025.1689103

**Published:** 2025-12-02

**Authors:** Zhaoqi Zhang, Ying Wang, Wei Yin, Qingyang Lei, Yuqiao Fu, Yingzi Liang, Ruilei Li, Ke Li

**Affiliations:** 1 Key Laboratory of Melanoma Research, The Third Affiliated Hospital of Kunming Medical University, Yunnan Cancer Hospital, Peking University Cancer Hospital Yunnan, Kunming, China; 2 Cancer Center, Union Hospital, Tongji Medical College, Huazhong University of Science and Technology, Wuhan, China

**Keywords:** skin cutaneous melanoma, integrated stress response, prognostic mode, immune micro-environment, immunohistochemistry

## Abstract

**Background:**

Skin cutaneous melanoma (SKCM) is a highly invasive cancer with dismal prognosis. Integrated stress response (ISR) is associated with tumorigenesis and progression, but its relationship with SKCM prognosis is unclear. This research aimed to identify relevant prognostic genes for SKCM prognosis and treatment insights.

**Methods:**

Data were obtained from public databases. Differential expression and regression analyses were used to identify prognostic genes. Based on these genes and independent prognostic factors, risk models and nomograms were constructed to assess their clinical application potential in SKCM. Then, immune microenvironment changes in SKCM were explored according to risk-group grouping, providing a basis for stratified treatment decisions for SKCM patients. Finally, RT-qPCR was used to validate the results.

**Results:**

*DTL*, *DTX3L*, *KCNMB1*, *NDRG1*, *GPX2*, *DERL3* and *MBTPS2* were validated as prognostic genes. A risk model was constructed to classify patients into High Risk Group (HRG) and Low Risk Group (LRG), with high-risk SKCM patients having a higher mortality rate. A nomogram integrating clinical indicators was an effective SKCM survival prediction tool. The immune microenvironment differed significantly between risk groups, and most differentially infiltrated immune cells had higher infiltration levels in the Low Risk Group (LRG). Immunotherapy analysis suggested that the Low Risk Group (LRG) might benefit little from treatment, highlighting the need for stratified treatment of SKCM patients. RT-qPCR showed that prognostic genes were up-regulated in human melanoma cells compared to fibroblasts.

**Conclusion:**

The identification of ISR-featured prognostic genes and risk score stratification provide new insights into targeting SKCM and enhancing the efficacy of immunotherapy.

## 1 Introduction

Cutaneous melanoma (CM) represents a highly invasive type of cancer originating from the malignant transformation of melanocytes, the pigment - synthesizing cells in human skin (1). CM accounts for only 4% of skin cancers, but it contributes to 75% of skin cancer-related deaths. Studies have demonstrated that genetic susceptibility, together with environmental exposure factors (mainly long-term ultraviolet radiation (UVR)) contribute to the occurrence and development of CM, among which (2). Statistics indicate that over 300,000 new cases of melanoma were diagnosed globally in 2020. The number of new melanoma cases is projected to increase by approximately 50% without effective intervention (3). While surgical resection is generally effective for early-stage CM, managing advanced cases presents substantial clinical challenges. Despite significant progress in the treatment using BRAF/MEK inhibitors and immune checkpoint inhibitors, many patients still develop therapeutic resistance due to tumor heterogeneity and adaptation (4, 5). Therefore, further exploration of new therapeutic targets and biomarkers is crucial for improving the prognosis of CM and providing novel clinical treatment strategies.

The Integrated Stress Response (ISR) is an adaptive mechanism activated by diverse stressors, including infection, hypoxia, nutrient deprivation, increased reactive oxygen species (ROS), endoplasmic reticulum stress and oncogene activation. It supports cellular resilience by promoting recovery and stability during exposure to severe stimuli and adverse conditions (6). Currently, the ISR is more and more recognized as a determining factor in tumor progression and development (7). In the tumor micro-environment, sustained stress conditions (such as hypoxia, nutrient deprivation, or oxidative stress) can lead to persistent activation of the ISR. This, in turn, promotes adaptive survival, metastasis, and drug resistance in tumor cells by regulating protein synthesis, metabolic reprogramming, and cell survival signaling pathways. For example, core ISR effector molecules like *ATF4* and *GCN2* can upregulate the expression of pro-survival genes, enabling tumor cells to survive under harsh conditions while potentially indirectly suppressing anti-tumor immune responses (8). Despite these advances, the precise role of ISR in melanoma, its crosstalk with oncogenic drivers and the tumor micro-environment remains poorly understood, underscoring the need for further investigation.

In this study, we utilized public databases and Integrated Stress Response-Related Genes (ISR-RGs) to identify genes linked to the prognosis of Skin Cutaneous Melanoma (SKCM) and the correlation between SKCM and ISR through multiple analytical methods. We then constructed a prognostic model with excellent predictive performance. This model evaluated the prognostic value of ISR-RGs in SKCM and explored the potential molecular regulatory mechanisms of prognostic genes, providing new insights for clinical decision-making and helping improve the prognosis of SKCM.

## 2 Method

### 2.1 Data sources

The TCGA-SKCM was acquired from The Cancer Genome Atlas (TCGA) database and encompassed 457 SKCM tissue samples (SKCM group). Meanwhile, the training set GSE15605 (platform: GPL570) was obtained from the Gene Expression Omnibus (GEO) database, included 58 SKCM tissue samples and 16 normal tissue samples. The validation set GSE19234 (platform: GPL570) was acquired from the same database, and the 44 SKCM tissue samples were included. Moreover, according to the previous report (9), a total of 529 ISR-related genes (ISR-RGs) after duplication removal were acquired (**Supplementary Table 1**). The screening strategy for ISR-RGs in this study targeted five key pathways, namely heat shock response (HSR), oxidative stress response (OSR), hypoxia stress response (HySR), DNA damage response (DDR), and unfolded protein response (UPR). It first initially screened pathway-related genes by matching the biological functions of each pathway to corresponding terms in the Gene Ontology (GO) database and retaining genes with an expression rate > 30% in at least one cell type; then refined the gene set by incorporating core regulatory factors and their target genes of each pathway as well as classic stress-related genes; finally verified the reliability of the screened gene set through cell-level stress score quantification via single-cell RNA sequencing, spatial distribution verification of the tumor microenvironment (TME) using spatial transcriptome data, and detection of gene expression changes in the cell model of pancreatic stellate cells (PSCs)-induced cancer-associated fibroblast (iCAF) differentiation.

### 2.2 Identification of candidate genes, and their functional enrichment and protein level interaction analyses

In the GSE15605 dataset, DEGs between the SKCM group and the control group were screened using the limma software package (version 3.56.2) (10) (*p* < 0.05, |log2FC| > 0.5) (11, 12). Additionally, significantly up- and down-regulated DEGs were visualized using volcano plots and heatmaps. Finally, candidate genes were identified from the overlap of DEGs and ISR-RGs using the ggvenn package (version 0.1.9) (13).

Moreover, to further understand the pathways involved in the candidate genes, the clusterProfiler package (v 4.8.2) (14) was applied to conduct GO and KEGG analyses of candidate genes (adjusted *p* < 0.05). The top 10 marked entries for each section of GO were visualized, as well as top 5 the marked KEGG pathways were visualized. Furthermore, to study the interactions among candidate genes at the protein level, the candidate genes were imported into the STRING database. After removal of isolated proteins, PPI (the protein-protein interaction) network was created via the Cytoscape software (v 3.9.1) (confidence level > 0.7) (15).

### 2.3 Recognition of prognostic genes and construction of the risk models

Proportional hazards (PH) hypothesis testing (*p*>0.05) and univariate Cox regression analysis (*p* < 0.01) were performed using the survival analysis software package (v 3.5-3) (16) to identify prognostic genes, and the Rbase function was used to plot a forest plot to visualize the results. Subsequently, LASSO regression analysis was executed via the glmnet package (v4.1-4) (17). When the model error rate was lowest, the coefficients of candidate gene variables that were not penalized to zero were selected as potential prognostic genes. Multivariate regression analysis was then used to determine the prognostic genes. Subsequently, the risk scores for each prognostic gene were calculated using the surv_cutpoint function to construct a risk model, where the coefficients represent the risk values corresponding to each prognostic gene, and the expression levels reflect the expression levels of each prognostic gene.


$$risk\;score=\sum_{i=1}^{n}coef(gene_{i})\times \exp r(gene_{i})$$

N represents the 7 prognostic genes included in the risk scoring formula, Coef represents the risk coefficient of each gene, and expr represents the expression level of each gene.

Subsequently, TCGA-SKCM samples were divided into HRG and LRG groups based on the optimal threshold, and survival curves were plotted. Additionally, Kaplan-Meier (KM) curves were plotted using survival analysis software to compare survival status differences between the two groups (*p* < 0.05). Next, the pROC software package (v 1.18.0) (18) was used to plot receiver operating characteristic (ROC) curves for 3-, 5-, and 7-year data to appraise the predictive performance of the model (AUC>0.6), and the results were further validated in GSE19234.

### 2.4 Relationship between clinical indicators and risk models and risk scores

So as to find out whether there was a difference between different clinical indicator subgroups in risk groups, TCGA-SKCM samples were grouped according to different clinical indicators. Specifically, into greater than 60 years old, less than 60 years old, M0, M1, N0 & N1, N2 & N3, T0 & T1 & T2, T3 & T4, Stage I & Stage II, Stage III & Stage IV. Subsequently, the survminer package (v 0.4.9) (19) was applied to perform log-rank tests (*p* < 0.05) and plot KM curves for visualizing the results. Moreover, based on the above groupings, the ggpubr package (v 0.6.0) (20) was adopted to assess the discrepancies in risk scores within the groupings (*p* < 0.05). In addition, to verify the clinical therapeutic guiding value of the ISR-related risk model, this study used the treatment annotation data of the TCGA-SKCM cohort to conduct subgroup survival analysis.

### 2.5 Independent prognostic analysis and construction of nomogram

For better application, in the TCGA-SKCM, risk score was integrated with clinical indicators to generate nomogram associated with survival of SKCM patients. First, univariate Cox regression analysis was conducted with survival analysis software packages for six variables, including risk scores and age (*p* < 0.05), and a PH hypothesis testing model was built (*p*>0.05). Multivariate Cox regression analysis was used to screen for independent prognostic factors, and the rms software package (v 6.5-0) (21) was used to construct an SKCM survival prediction nomogram. Finally, the nomogram was appraised through plotting the calibration curve, and the survivalROC package (v 1.0.3.1) (22) was employed to plot the ROC curves to appraise the precision of the model.

### 2.6 Gene set enrichment analysis, chromosome localization analysis and protein expression distribution analysis

To investigate the latent functions of the prognostic genes in SKCM, based on the GSE15605, the cor function was applied in GSE15605 to analyze the associations among each prognostic gene and other genes, and the list of genes was generated by sorting the genes according to the correlations from the largest to the smallest. The backdrop gene set “C2: KEGG” was retrieved from the MSigDB, the clusterProfiler package was applied to conduct the GSEA (adjusted *p* < 0.05). Furthermore, the RCircos package (v 1.2.2) (23) was employed to acquire the distribution of prognostic genes on chromosomes, supplying a basis for subsequent applications of prognostic genes. Finally, to understand the expression of proteins encoded by prognostic genes in different tissues and organs, the HPA database (https://www.proteinatlas.org/) was utilized to analyze the differences in the distribution of prognostic genes in immunohistochemical images of SKCM tumor tissues and paraneoplastic control tissues. To explore the differences in biological functions associated with prognostic genes and the signaling pathways they are involved in, based on the TCGA-SKCM training set, the cor function from the Rbase package was used to calculate the Spearman correlation coefficient between each prognostic gene and all other genes, respectively. According to these coefficients, the genes were sorted in the order from the most positively correlated to the most negatively correlated. Subsequently, the R package “clusterProfiler” was adopted to perform GSEA. In this study, the “c5.go.v7.5.1.symbols.gmt” gene set was used as the background gene set. Via the GSEA function in R, enrichment analysis was conducted on the sorted gene list within the background gene set (with the criteria of adj.p < 0.05 and |NES| > 1).

### 2.7 Immune infiltration analysis and immunotherapy response

To investigate the changes of immune characteristics after risk stratification, the xcell package (v 1.1.0) (24) was applied to assess the infiltration of 64 immune cells. Subsequently, differentially infiltrated immune cells between risk groups were obtained by ggpubr package (v 0.6.0) and Wilcoxon test. Furthermore, the cor function was employed to further understand the Spearman correlations between differentially infiltrated immune cells and prognostic genes (|cor| > 0.3, *p* < 0.05). Moreover, the ESTIMATE algorithm (v 1.0.13) (25) was deployed to compute the immune, stromal, and ESTIMATE scores for each SKCM sample, and the differences between HRG and LRG were likened (*p* < 0.05). Afterwards, to appraise the immunotherapy response in SKCM samples, the TIDE website (http://tide.dfci.harvard.edu/) was adopted to assay the sensitivity of the risk model to immunotherapy and visualized by the score (including Immune Dysfunction, Immune Exclusion, and TIDE total scores). Finally, the Wilcoxon test was applied to compare the differences in TIDE scores between HRG and LRG (*p* < 0.05), and the cor function was exploited to analyze the relative relationship between risk scores and TIDE scores.

### 2.8 Drug sensitivity analysis and mutation analysis

To comprehend the expression of prognostic genes within 42 immune checkpoints (26), firstly, differential immune checkpoints between risk groups were gained (*p* < 0.05), subsequently, the relatedness between differential immune checkpoints and risk scores was analyzed. The ggplot2 package (v 3.3.6) was applied to visualize the 2 most highly correlated results (|cor| ≥ 0.3, *p* < 0.05). Besides, data from the v2 version of the GDSC database (https://www.cancerrxgene.org/) were employed as a training set, and the oncoPredict package (v 1.2) (27) was applied to construct a model that predicted the half maximal inhibitory concentration (IC_50_) values of the drugs for the SKCM samples. Subsequently, the differences in half-inhibitory concentration values of each drug in HRG and LRG and their correlation with risk scores were analyzed (|cor| ≥ 0.3, *p* < 0.05). Finally, the oncoplot function of the maftools software package (v 2.14) (28) was applied to generate a waterfall plot to explore the variation characteristics of HRG and LRG.

### 2.9 Construction of regulatory network

To investigate the possible expression regulation patterns of prognostic genes, the miRDB (https://mirdb.org/) was applied to predict microRNAs (miRNAs) targeting prognostic genes. Then, the miRNAs were sorted according to likelihood scores, and miRNAs from the top10 of each prognostic gene were employed as visualization elements. Subsequently, the starBase database (https://rnasysu.com/encori/)was applied to forecast the long noncoding RNAs (lncRNAs) of miRNAs (pancancerNum≥10), and the top5 lncRNAs sorted according to pancancerNum were employed as visualization elements. The Cytoscape software was applied to visualize the mRNA-miRNA-lncRNA network. Moreover, to identify the transcription factors (TFs) that had interactions with prognostic genes, the CistromeDB (http://dbtoolkit.cistrome.org/) was applied to predict the TFs targeting prognostic genes.

### 2.10 Reverse transcription quantitative PCR

To confirm the consistency of prognostic gene expression with bioinformatics results, the prognostic gene expression was verified by RT-qPCR. The human malignant melanoma cell lines A375 (CL-0014) and SK-MEL-28 (CL-0717) were purchased from Pricella (China), while MV3 (iCell-h462) was acquired from Icell (China). Fibroblasts (ORC0493) were obtained from Aoruicell (China). The cell lines were resuscitated in 89% MEM (Meilunbio, MA0217, China) and 89% DMEM medium (Meilunbio, MA0212-2, China) containing 10% fetal bovine serum (FBS; Procell, 164210, China), 1% penicillin, and 1% streptomycin (Meilunbio, MA0110-1, China). Cells were passaged using complete medium under conditions of 37 °C and 5% CO2. Three cases of each of the human melanoma cell lines (A375, SK-MEL-28, MV3) and fibroblasts were used to extract their total RNAs using the Trizol method (Vazyme, R401-01, China) (29), and the cDNA was gained using the Hifair^®^III 1st Strand cDNA synthesis supermix for qPCR (gdna digester plus) (Yisheng, 11141ES60, China). Finally, RT-qPCR was executed using ^®^ 2 × Universal Blue SYBR Green qPCR Master Mix kit (Servicebio, G3326-05, China). GAPDH was employed as housekeeping gene and the 2-ΔΔCt (30) was employed to compute prognostic gene expression. Primer sequences were shown in **Table 1**. The resultant data were statistically analyzed and visualized by Graphpad Prism (v8.0) (31).

**Table 1:** Primer sequences of housekeeping genes and 7 key prognostic genes.

Genes	Forwardprimer	Reverseprimer
DTX3L	TTGACGAAAAACCTGTGCCC	ACCAGACGGTGTTTCTGCTT
GPX2	ACTTCACCCAGCTCAACGAG	ATGCTCGTTCTGCCCATTCA
KCNMB1	GGAACGAAACCAGCGTCCTA	GGATGGCTCTACTTCTGGGC
DERL3	CTCGGGTGAGGGTCAACTTC	TCTGAAGTCCCAGGAAGCCA
NDRG1	AGCTCGTCAGTTCACCATCC	AGTCTCGATGTCCTGCTCCT
DTL	GGAGTTGGAGGCGATAACGA	GCATCAGGGTCGGAGGAAAA
MBTPS2	GATCTGCCAGTGGTTGTGGA	ACCGTGCTGTAACCATCCAG
GAPDH	ATGGGCAGCCGTTAGGAAAG	AGGAAAAGCATCACCCGGAG

### 2.11 Statistical analysis

The R software (v 4.2.2) was applied to proceed bioinformatics analysis. The Wilcoxon test and t-test were utilized to evaluate paired sample comparisons, *p < 0.05.

### 2.12 Ethics statement

Ethics approval and consent to participate not applicable. All data were downloaded from the internet.

## 3 Results

### 3.1 Identification and functional profiling of candidate genes with ISR features in SKCM

**Figure 1:**
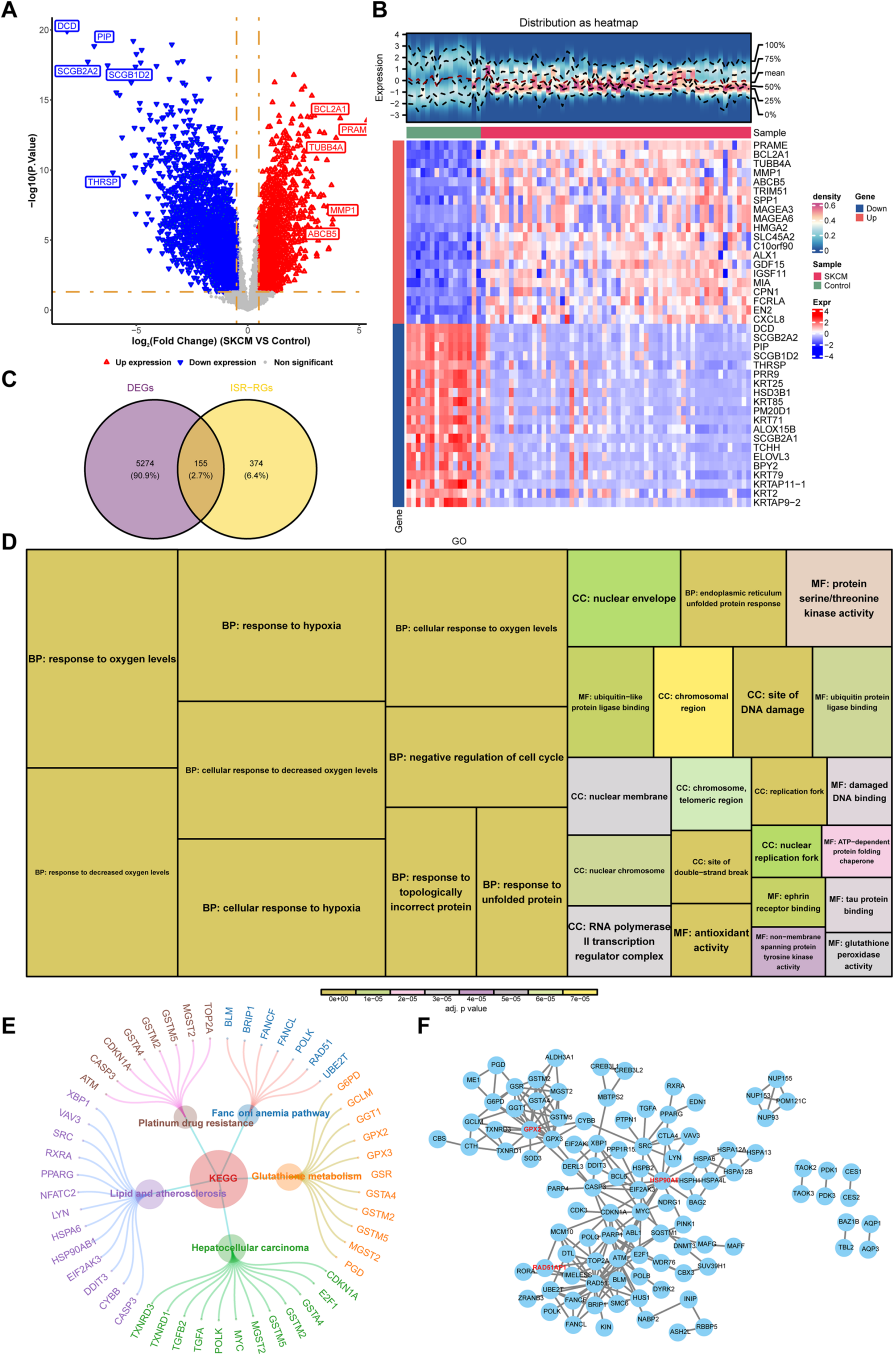
The profile of differentially expressed genes (DEGs) and ISR-RGs. **(A)** Volcano plot of differential gene expression. Each dot represents a gene, with red indicating significantly up-regulated genes, blue indicating significantly down-regulated genes, and gray representing non-significant genes. **(B)** Heatmap of differentially expressed genes. The upper section shows the gene distribution heatmap, while the lower section displays the gene expression heatmap. The horizontal axis represents samples, and the vertical axis represents differentially expressed genes. Colors indicate standardized gene expression levels, with red representing high expression and blue representing low expression. **(C)** Venn diagram of the overlapped genes between DEGs and ISR-RGs. **(D)** GO enrichment analysis of candidate genes. Show top 10 pathways. The area size represents the number of genes, and the color indicates significance. **(E)** KEGG enrichment analysis, displaying the top 5 pathways. Circles represent pathways, outer circles represent genes. **(F)** PPI network analysis of 155 candidate genes.

The 5,429 DEGs were identified between SKCM and control samples, including 2,707 up-regulated and 2,722 down-regulated genes in SKCM samples (**Figure 1A**). The heatmap showed that the 40 DEGs with the largest FCs (both up-regulated and down-regulated) exhibited distinct expression profiles between control and SKCM samples (**Figure 1B**). In SKCM, 155 candidate genes with ISR features were obtained through the intersection of DEGs and ISR-RGs (**Figure 1C**). Subsequently, the candidate genes were markedly enriched in 1,482 GO entries (adjusted *p* < 0.05) (**Supplementary Table 2**). Specifically, there was a marked enrichment mainly in negative regulation of cell cycle (BP), site of DNA damage (CC) and antioxidant activity (MF) (**Figure 1D**). Moreover, candidate genes were markedly enriched in 64 KEGG pathways (adjusted *p* < 0.05) (**Supplementary Table 3**), including glutathione metabolism, hepatocellular carcinoma, and lipid and atherosclerosis (**Figure 1E**). In addition, the PPI network of candidate genes contains 103 proteins and 213 interaction relation pairs, among which *RAD51*, *HSP90AB1* and *GPX2* had many interactions with other candidate genes (**Figure 1F**).

### 3.2 Construction and validation of ISR-related risk model for SKCM survival

**Figure 2:**
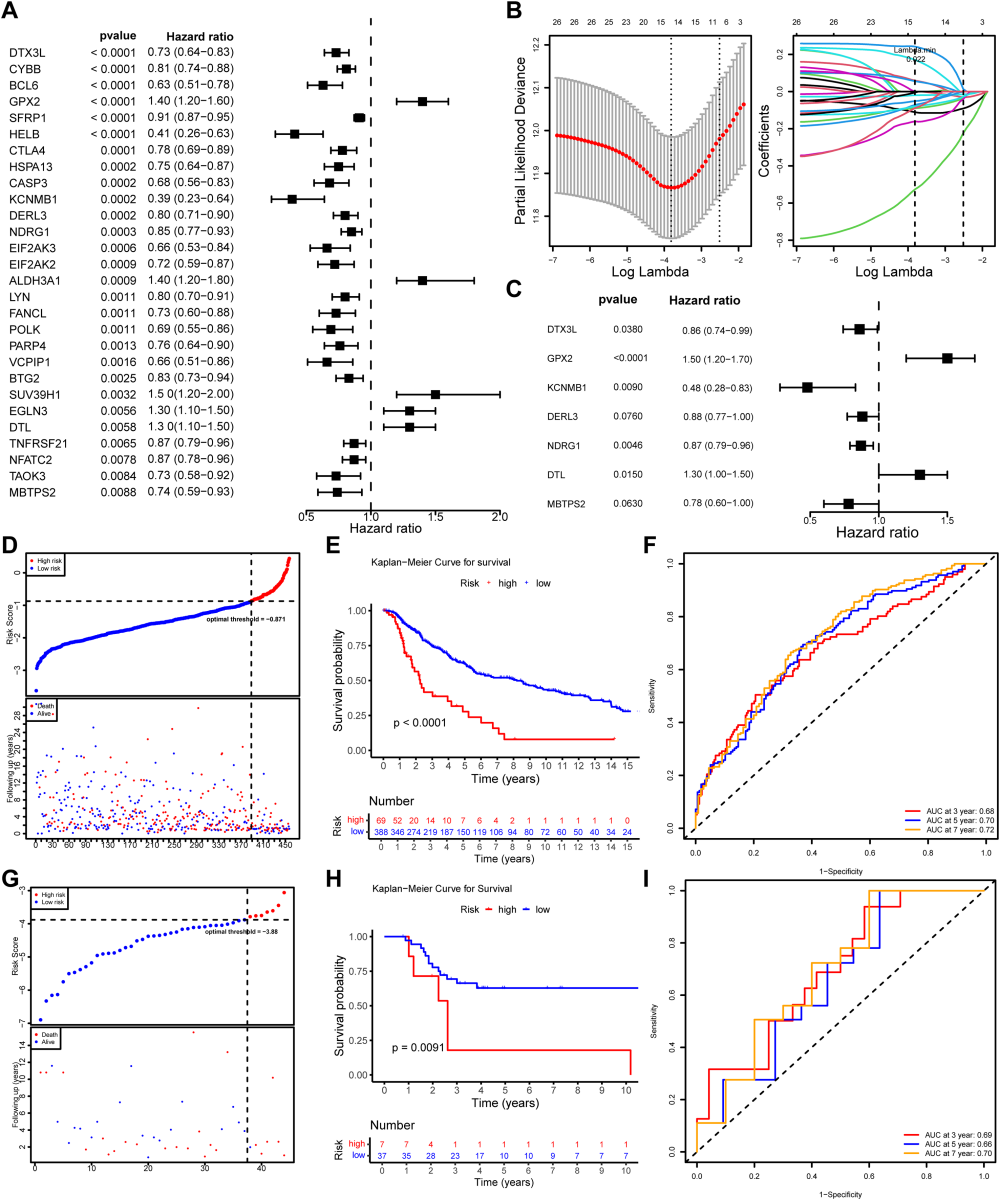
Screening key ISR-RGs to construct a SKCM prognostic model. **(A)** Univariate Cox regression forest plot. **(B)** LASSO regression analysis plots: the left panel shows the trajectory of coefficients against the penalty parameter, and the right panel displays the cross-validation error curve for selecting the optimal penalty parameter. **(C)** Multivariate Cox regression forest plot. **(D)** Risk score distribution plot for the TCGA-SKCM training cohort. **(E)** Kaplan-Meier survival curve comparing high-risk and low-risk groups in the TCGA-SKCM training cohort. **(F)** Time-dependent ROC curves for 3-year, 5-year, and 7-year survival predictions in the training cohort. **(G-I)** Risk curves, Kaplan-Meier survival curves and ROC curves in the validation set (GSE19234).

To construct a robust prognostic model, we first performed the proportional hazards (PH) assumption test on 155 candidate genes, among which 142 genes satisfied the model assumptions (p > 0.05) (**Supplementary Table 4**). Based on the univariate Cox regression analysis of these 142 genes, 28 candidate genes that were significantly associated with the overall survival of SKCM patients were identified (p < 0.01) (**Figure 2A**; **Table 2**). Subsequently, 15 LASSO-feature genes were identified (lambda. min = 0.022), including *DTX3L*, *BCL6*, *GPX2*, *SFRP1*, *HELB*, *CASP3*, *KCNMB1*, *DERL3*, *NDRG1*, *EIF2AK2*, *ALDH3A1*, *FANCL*, *EGLN3*, *DTL* and *MBTPS2* (**Figure 2B**). Then, 7 genes (*DTX3L*, *GPX2*, *KCNMB1*, *DERL3*, *NDRG1*, *DTL* and *MBTPS2*) passed the multivariate Cox regression analysis, and were identified as prognostic genes (**Figure 2C**). Then, a risk model was constructed by calculating risk scores for prognostic genes (risk score = *DTX3L* × -0.153 + *GPX2* × 0.378 + *KCNMB1* × -0.725 + *DERL3* × -0.125 + *NDRG1* × -0.138 + *DTL* × 0.230 + *MBTPS2* × -0.248) and categorizing SKCM samples into HRG (n = 69) and LRG (n = 388) based on the optimal threshold (C ut-off: -0.871) of the scores. The results displayed an escalation in the death count with the rising risk score (**Figure 2D**). The KM curve shows that there is a significant difference in survival rates between HRG and LRG (*p* < 0.0001), with HRG having a lower survival rate (**Figure 2E**). AUCs for 3-, 5-, and 7-year survival were 0.68, 0.70, and 0.72, respectively, indicating that the risk model built based on prognostic genes exhibits strong predictive capacity for SKCM survival (AUC > 0.6) (**Figure 2F**). In the GSE19234 dataset, the SKCM were categorized into HRG (n = 7) and LRG (n = 37) after the optimal threshold (-3.88) of the scores. The remaining results were consistent with those from TCGA-SKCM, confirming the credibility of the risk score model built based on *DTX3L, GPX2, KCNMB1, DERL3, NDRG1, DTL*, and *MBTPS2* (**Figures 2G–I**).

**Table 2:** PH hypothesis test results for 27 candidate genes.

Gene	P-value
DTX3L	0.100
CYBB	0.302
BCL6	0.256
GPX2	0.766
SFRP1	0.475
HELB	0.089
HSPA13	0.663
CASP3	0.863
KCNMB1	0.963
DERL3	0.781
NDRG1	0.321
EIF2AK3	0.147
EIF2AK2	0.088
ALDH3A1	0.622
LYN	0.485
FANCL	0.284
POLK	0.693
PARP4	0.475
VCPIP1	0.355
BTG2	0.271
SUV39H1	0.149
EGLN3	0.778
DTL	0.583
TNFRSF21	0.748
NFATC2	0.068
TAOK3	0.210
MBTPS2	0.549

Treatment regimens with extremely small sample sizes (hormone therapy, n=5; adjuvant therapy, n=6) and samples with missing treatment information (n=1) were excluded, and subgroup survival analysis was conducted using treatment annotation data from the TCGA-SKCM cohort (n=255); among these, the chemotherapy subgroup included 117 samples with complete survival information, with an optimal cutoff value of -1.35, and Kaplan-Meier (KM) analysis showed that the survival of the high-risk group (HRG) was significantly worse than that of the low-risk group (LRG) (log-rank p<0.05) (**Supplementary Figure 1A**); the immunotherapy subgroup included 86 samples, with an optimal cutoff value of -1.57, and no statistically significant difference in survival was observed between the two groups (log-rank p>0.05) (**Supplementary Figure 1B**), which was speculated to be related to the heterogeneity of the tumor microenvironment and required further verification with an expanded sample size; the vaccine therapy subgroup included 28 samples, with an optimal cutoff value of -1.83, and no significant difference in survival was found between the two groups (log-rank p>0.05) (**Supplementary Figure 1C**), which might be limited by the small sample size; the targeted molecular therapy subgroup included 18 samples, with an optimal cutoff value of -1.71, and no significant survival difference was observed between the two groups (log-rank p>0.05) (**Supplementary Figure 1D**), which was speculated to be related to the fact that the response to targeted therapy depends more on specific gene mutation status (rather than the expression of ISR-related genes) or insufficient sample size leading to limited statistical power; in summary, the ISR-related risk model has a clear predictive value for the survival prognosis of patients receiving chemotherapy and can assist in screening patients who may benefit from chemotherapy, while its predictive efficacy for immunotherapy, vaccine therapy, and targeted molecular therapy still requires further verification in larger sample size cohorts, providing directions and boundaries for the clinical translation of the model.

### 3.3 ISR-related risk score was positively associated with clinical progression in SKCM patients

**Figure 3:**
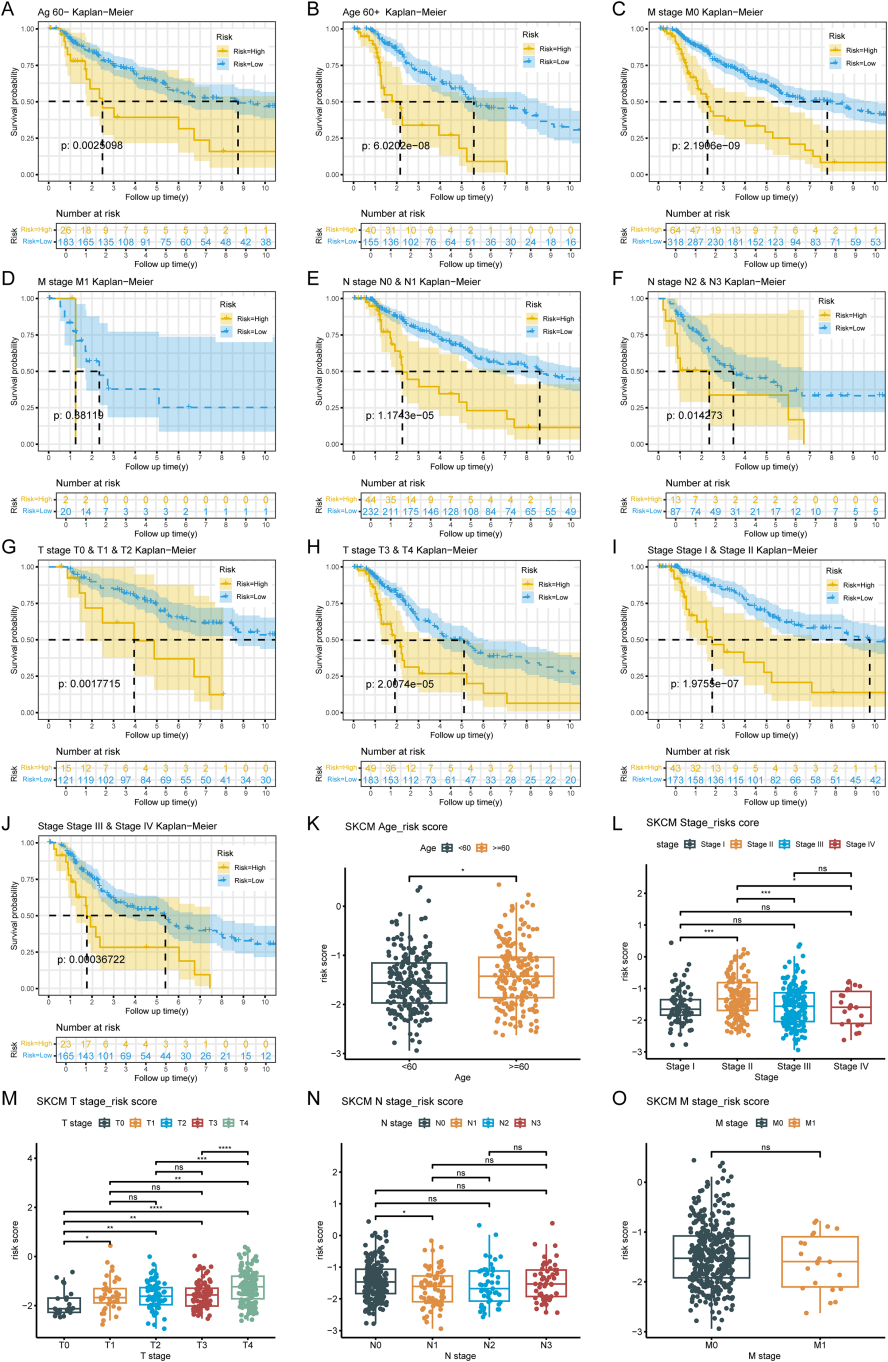
Analysis of the correlation between risk scores and clinical characteristics. **(A-J)** Survival analysis across clinical subgroups. The lower portion of each plot shows the number of patients at risk over time, while the upper portion displays the Kaplan-Meier survival curves. Each panel represents a different clinical subgroup: **(A)** Age < 60 years; **(B)** Age ≥ 60 years; **(C)** M0; **(D)** M1; **(E)** N0 & N1; **(F)** N2 & N3; **(G)** T0, T1 & T2; **(H)** T3 & T4; **(I)** Stage I & Stage II; **(J)** Stage III & Stage IV. (**K-O**) Boxplots of risk scores stratified by clinical features. The x-axis represents different clinical subgroups, and the y-axis shows the risk score. Statistical significance is indicated above each comparison: ns means not significant, * means p < 0.05, ** means p < 0.01, *** means p < 0.001, and **** means p < 0.0001.

The analysis results based on subgroups of different clinical indicators showed that, except for the M1 subgroup (p=0.38119), there were significant differences in survival rates among risk groups across all subgroups. It was noteworthy that patients in the LRG generally exhibit better survival prognosis than those in the HRG, which further validates the reliability of the risk model (**Figures 3A–J**). The risk score significantly increased with patient age and T stage, with significant differences observed in multiple T stage (*p* < 0.05). Additionally, statistically significant differences were found between different N/M stage groups (e.g., Stage I vs. Stage II, N0 vs. N1) (*p* < 0.05). The above results suggested the clinical value of the ISR-related risk score for personalized treatment in patients with cutaneous melanoma (**Figures 3K–O**).

### 3.4 Nomogram integrating the risk score and N stage with favorable SKCM survival predictive accuracy

**Figure 4:**
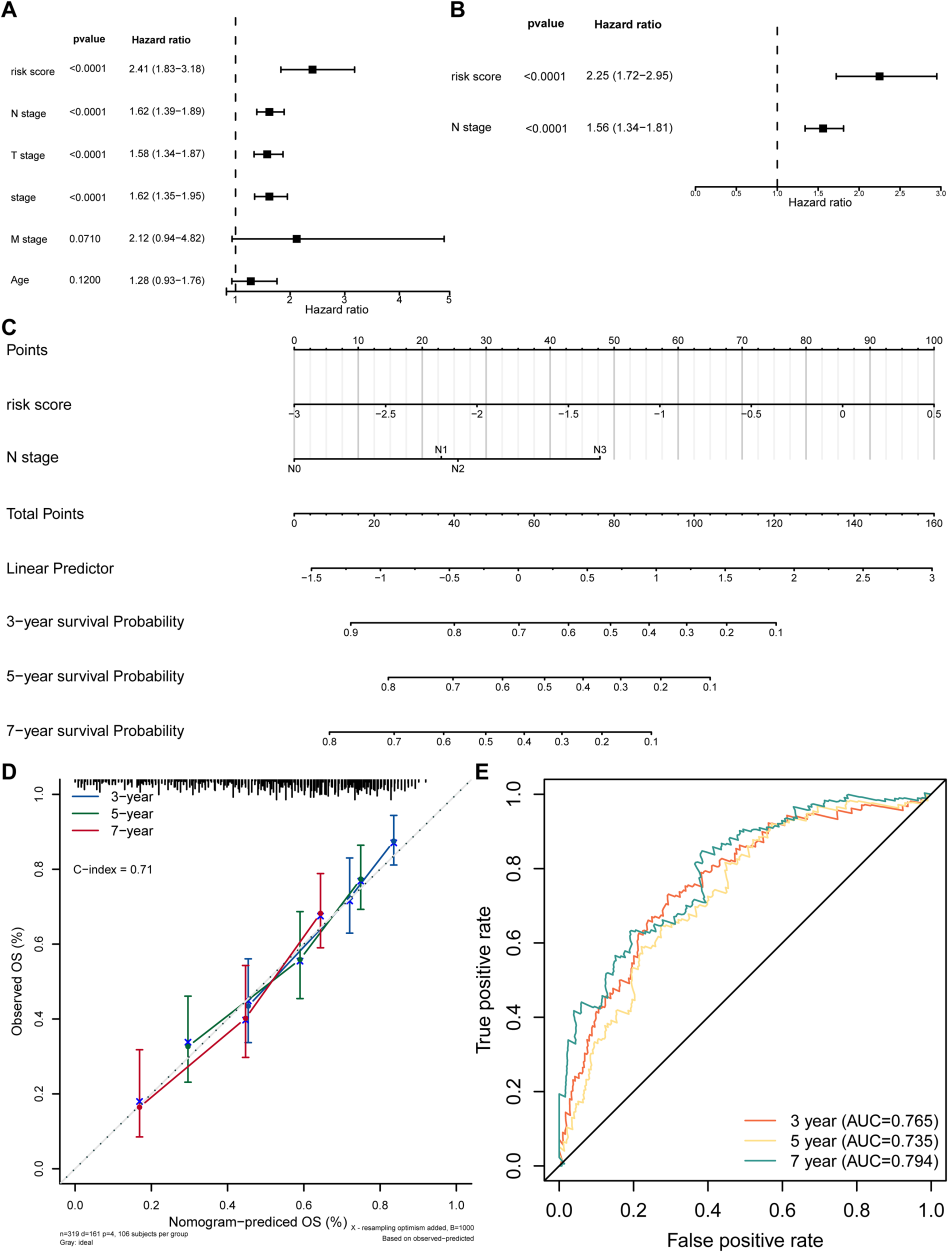
Independent prognostic analysis. **(A)** Forest plot of univariate Cox regression for independent prognosis. **(B)** Forest plot of multivariate Cox regression for independent prognosis. **(C)** Nomogram for predicting survival based on independent prognostic factors. **(D)** Calibration curve of the nomogram. **(E)** ROC curve of the nomogram model.

By using Cox regression analysis, the N stage and risk score were recognized as independent prognostic factors after analysis (**Figures 4A, B**; **Table 3**). For better application, a nomogram predicting survival for SKCM was constructed. The nomogram showed that higher total points corresponded to decreased 3-, 5-, and 7-year survival rates in SKCM patients (**Figure 4C**). Subsequently, the calibration curve displayed that the predicted values were not markedly different from the actual values (C-index = 0.71), and the AUCs were 0.765, 0.735 and 0.794 of 3,5,and 7 years, proving the beneficial precision of the nomogram (AUC > 0.7) (**Figures 4D, E**). The above findings also provide a potent tool for clinical application in SKCM patients.

**Table 3:** Results of PH hypothesis test between N stages and risk score.

Gene	P-value
risk score	0.880
N stage	0.391

### 3.5 Critical pathway analysis of the prognostic genes and their expression in cutaneous melanoma tissues

**Figure 5:**
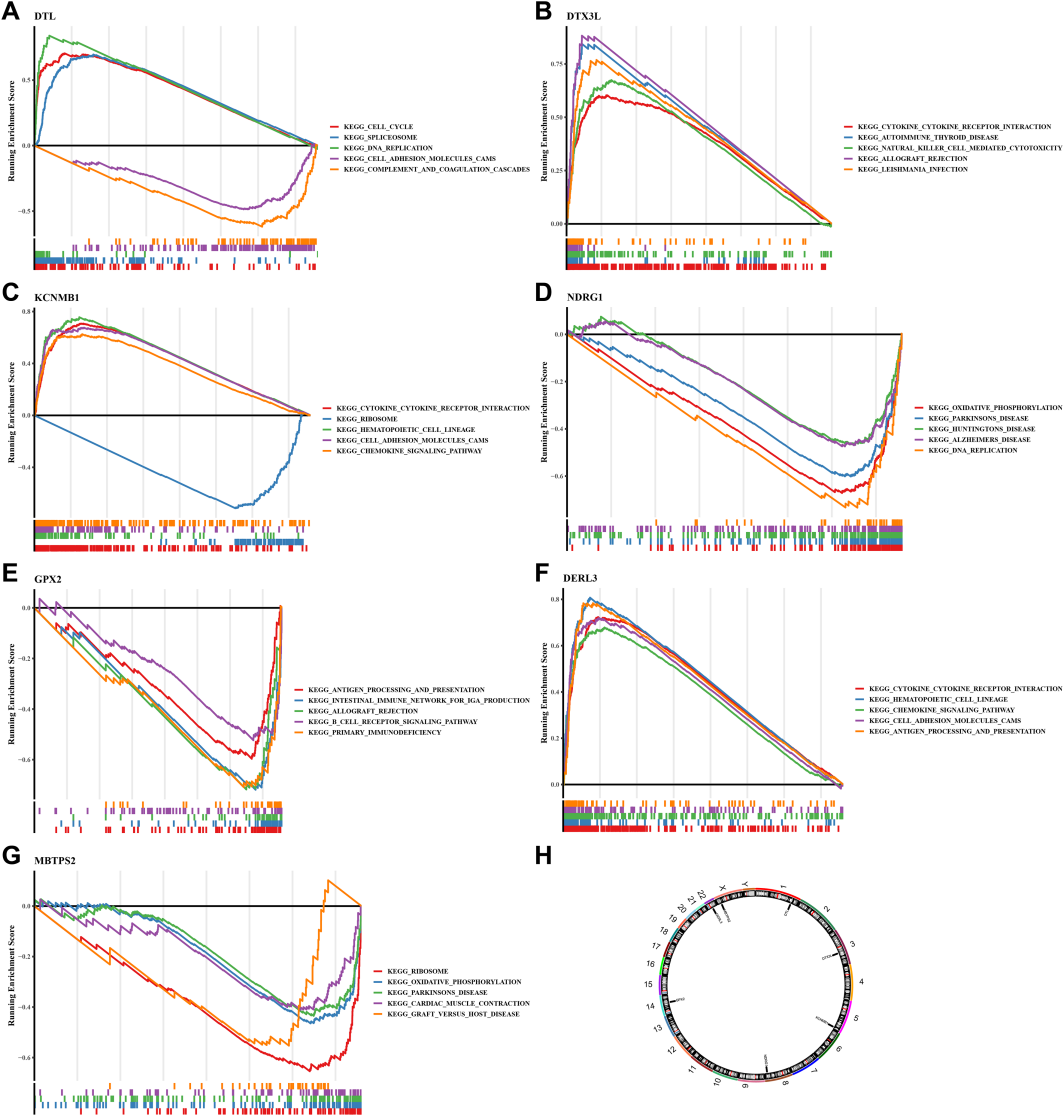
GSEA analysis, chromosomal localization and expression profiles of protein.**(A)** GSEA analysis of *DTL*; **(B)** GSEA analysis of *DTX3L*; **(C)** GSEA analysis of *KCNMB1*; **(D)** GSEA analysis of *NDRG1*; **(E)** GSEA analysis of *GPX2*; **(F)** GSEA analysis of *DER3L*; **(G)** GSEA analysis of *MBTPS2*. **(H)** Chromosomal distribution of prognostic genes. The figure is divided into three parts. The top part shows the calculation process of the enrichment score (ES) value. For each gene from left to right, an ES value is calculated and connected in a line. On the far left, there is a particularly obvious peak, which is the ES value on the gene set phenotype. Each line in the middle part of the figure represents a gene in the gene set and its ranking position in the gene list. The bottom part shows the matrix of the association between genes and phenotypes.

The ISR-related prognostic model exhibits outstanding prognostic performance in SKCM. To clarify the molecular regulatory mechanisms underlying the seven key prognostic genes, further investigation is necessary. Functional characterization results showed that *GPX2*, *DERL3* and *MBTPS2* were markedly enriched in 52, 34, 72, 44, 42, 41, and 16 GO terms (p.adjust<0.05), respectively (**Supplementary Table 4**). The top five pathways with the highest enrichment degree of each gene are shown in **Figures 5A–G**). Among them, *DTX3L*, *KCNMB1* and *DERL3* were co-enriched in cytokine-cytokine receptor interaction pathway. *KCNMB1* and *DERL3* were also co-enriched in chemokine signaling pathway. Notably, *GPX2* was markedly enriched for pathways such as primary immunodeficiency, suggesting its relevance to inflammation. In addition, *DTL* and *NDRG1* were co-enriched to pathways such as DNA replication, whereas *MBTPS2* and *NDRG1* were markedly enriched to pathways such as oxidative phosphorylation. Cytokines and chemokines serve as key molecules mediating immune cell recruitment, tumor cell proliferation, and metastasis. Notably, certain chemokines are closely associated with the pathology and prognosis of SKCM (32), highlighting the correlation between prognostic genes and SKCM pathogenesis. This also provides therapeutic targets for intervening in SKCM progression by targeting and regulating the activity of these critical pathways.

To further explore the potential roles of these seven prognostic genes in SKCM from a global functional perspective, we performed GSEA based on the TCGA-SKCM training set. The GSEA results revealed that each gene was significantly associated with a large number of biological processes (adj.p < 0.05 and |NES| > 1). Specifically, DERL3 was significantly enriched in 1304 pathways, DTL in 1687 pathways, DTX3L in 1103 pathways, GPX2 in 433 pathways, KCNMB1 in 2465 pathways, MBTPS2 in 395 pathways, and NDRG1 in 1250 pathways (**Supplementary Tables 5–11**). Among these enriched pathways, all prognostic genes were significantly enriched in stress response pathways closely related to the classical Integrated Stress Response (ISR). In detail, DERL3 was associated with the Stress-Activated Protein Kinase (SAPK) signaling cascade and osmotic stress regulation; DTL focused on stress granule assembly and the regulation of stress-induced translation initiation; DTX3L participated in SAPK signaling and stress translation regulation; GPX2 regulated endoplasmic reticulum stress response; KCNMB1 was involved in SAPK signaling and stress granule assembly; MBTPS2 was associated with endoplasmic reticulum stress, stress-induced translation initiation, and stress granule assembly; and NDRG1 participated in SAPK signaling and stress transcription regulation. These findings confirm that all seven prognostic genes establish functional associations through core or associated mechanisms of the classical ISR, indicating that they are not indirect response genes but functionally relevant molecules in the classical ISR signaling network.

**Figure 6:**
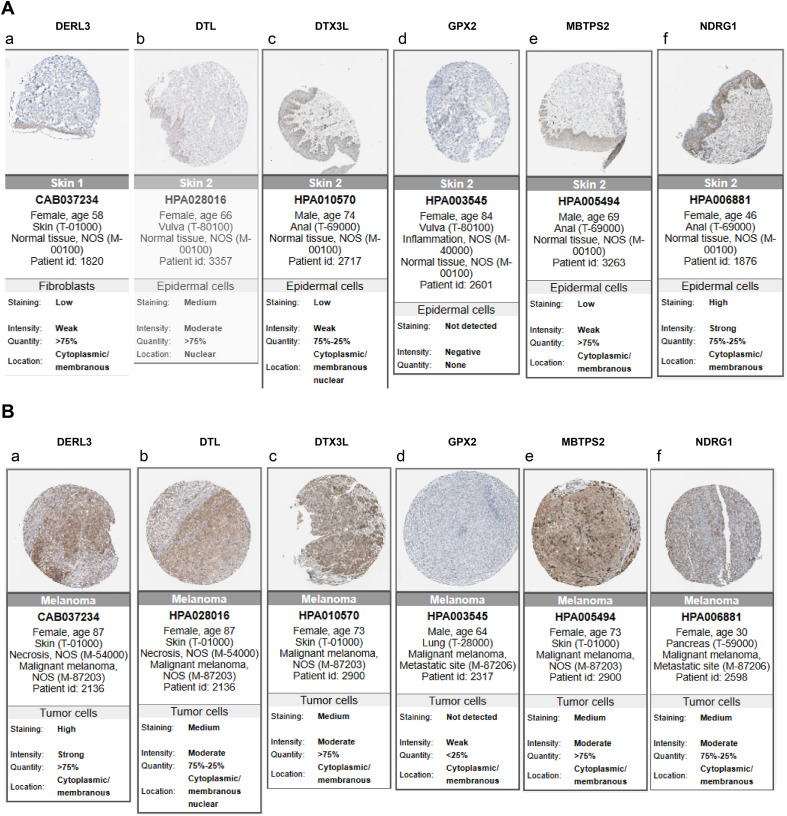
Analysis of protein expression distribution. **(A)** Protein expression levels of prognostic genes in normal tissues; **(B)** Protein expression levels of prognostic genes in SKCM tissues. Darker colors indicate higher expression levels.

Clarifying the specific location of prognostic genes on chromosomes helped to gain insight into the biological processes and signaling pathways in which prognostic genes might be involved, providing important clues for understanding the genetic basis and molecular mechanisms of SKCM. We found that the *DTL*, *DTX3L*, *KCNMB1*, *NDRG1*, *GPX2*, *DERL3* and *MBTPS2* were localized to chromosomes 1, 3, 5, 8, 14, 22 and X, respectively (**Figure 5H**). Then, we analyzed these genes by immunohistochemistry images in SKCM and normal tissue adjacent to the cancer. Results demonstrated that except for *GPX2*, the rest of the prognostic genes were highly expressed in SKCM tissues, especially *MBTPS2* (**Figures 6A,B**) (*KCNMB1* did not match related results). These results provided a basis for prognostic assessment of SKCM and for guiding therapeutic decisions.

### 3.6 Association of ISR-related genes with the immune microenvironment and immunotherapy response

**Figure 7:**
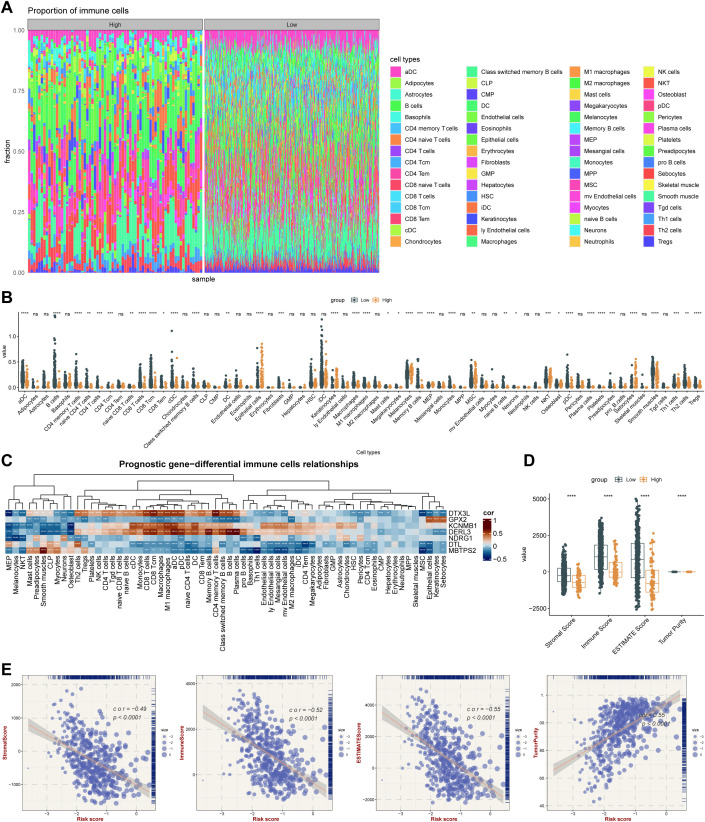
Integrated stress response-related prognostic model for TME analysis. **(A)** Immune cell infiltration proportion. The x-axis represents individual samples grouped by category, the y-axis shows the proportion of immune cell infiltration, and different colors indicate different cell types. **(B)** Differences in immune cell enrichment scores between high-risk and low-risk groups in the TCGA cohort. * means p < 0.05, ** means p < 0.01, *** means p < 0.001, **** means p < 0.0001; ns, not significant. **(C)** Correlation between key genes and immune cells. **(D)** ESTIMATE analysis results in the TCGA cohort. **** means p < 0.0001. **(E)** Correlation analysis between ESTIMATE metrics (Stromal Score, Immune Score, ESTIMATE Score, and Tumor Purity) and risk score in the TCGA cohort.

**Figure 8:**
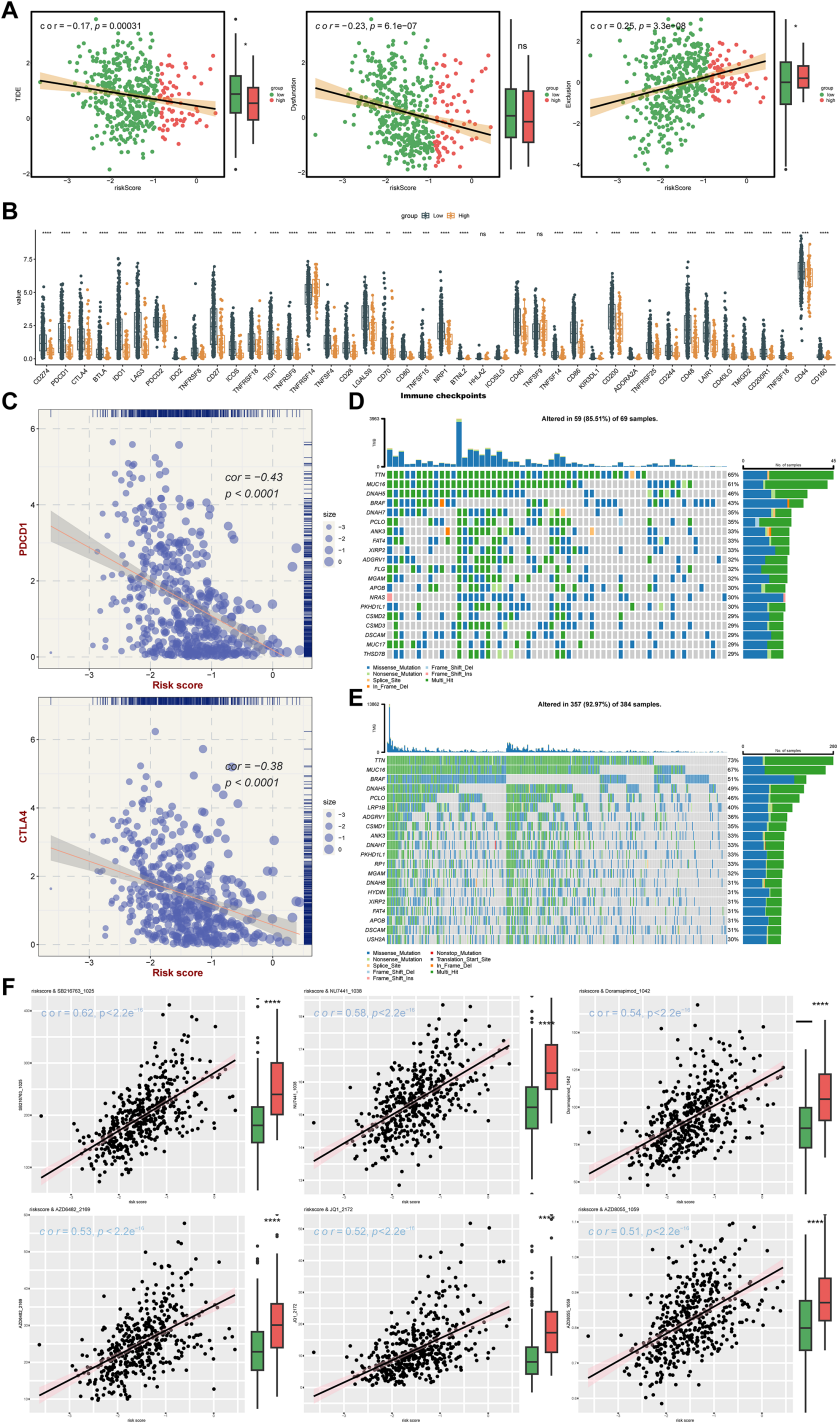
Analysis of therapy response. **(A)** TIDE algorithm risk score correlation analysis: The x-axis represents the risk score, while the y-axis corresponds to the TIDE score, immune dysfunction score, and immune exclusion score, with point color indicating different risk groups. **(B)** Immune checkpoint differential analysis: The x-axis shows different genes, the y-axis displays gene expression levels, and asterisks indicate statistical significance,*p means p < 0.05, **p means p < 0.01, *** means p < 0.001, **** means p < 0.0001. **(C)** Scatter plot of immune checkpoint and risk score correlation: The x-axis represents risk score, the y-axis shows gene expression, and point size reflects risk score magnitude. **(D)** High-risk group gene mutation waterfall plot: The y-axis shows genes, the x-axis represents samples, colors denote mutation types, and the right panel displays mutation type statistics and overall mutation rate. **(E)** Low-risk group gene mutation waterfall plot. **(F)** Drug sensitivity analysis.

Infiltration of immune cells between risk groups was displayed in **Figure 7A**, and the 38 differentially infiltrated immune cells were gained between risk groups. Notably, most differentially infiltrated immune cells exhibited higher infiltration levels in the LRG, whereas epithelial cells, mesenchymal stem cells (MSC), and natural killer T cells (NKT) showed elevated abundance in the HRG (**Figure 7B**). This suggested a possible reason for the poor prognosis of SKCM in HRG. Interestingly, *DTX3L*, *KCNMB1*, and *DERL3* demonstrated significant positive correlations with differentially infiltrated immune cells, whereas *GPX2*, *NDRG1*, *DTL*, and *MBTPS2* exhibited negative associations. Specifically, *MBTPS2* demonstrated the strongest negative correlation with T helper 1 (Th1) cells (r = -0.47, *p* < 0.0001), whereas *DERL3* showed the strongest positive link with plasma cells (r = 0.68, *p* < 0.0001) (**Figure 7C**) (**Supplementary Table 12****).** Subsequently, notable differences in immune, stromal, and ESTIMATE scores were identified between risk groups, with all scores being markedly elevated in the LRG. Tumor purity based on this inference was also markedly different within the risk groups (*p* < 0.0001) (**Figure 7D**), and there was a strong positive relativity between the 3 scores and tumor purity with the risk score (cor > 0.45, *p* < 0.0001) (**Figure 7E**). This had important implications for biological studies of SKCM and assessment of treatment response. Furthermore, the LRG had higher TIDE total score and immune dysfunction score. The LRG had markedly higher TIDE scores, suggesting that the tumors were more likely to have an immune escape profile, while the HRG had higher immune exclusion scores. Compared with low-risk patients, high-risk patients have a relatively milder state of immune suppression and may benefit more from immunotherapy (**Figure 8A**). Besides, 40 of the 42 immune checkpoints were markedly different in the risk group. And most of the differential immune checkpoints were highly expressed in the LRG, such as CD200, CD44 and CD48 (**Figure 8B**). The PDCD1 (cor = -0.43) and CTLA4 (cor = -0.38) showed a marked negative relativity with the risk score (*p* < 0.0001) (**Figure 8C**). Overall, alterations in the tumor immune microenvironment may be related to the expression status of ISR-related prognostic genes, leading to significant differences in immune status between HRG and LRG, which consequently affects the therapeutic benefits for patients subjected to immunotherapy. These results suggested that stratifying SKCM patients using the ISR-associated risk score effectively predicts immunotherapy response. This approach is crucial for achieving precision medicine, thereby improving therapeutic outcomes and prognosis.

### 3.7 Somatic mutation pattern and drug sensitivity altered by ISR-related risk scores

For optimizing personalized therapeutic strategies based on expression profiling of seven ISR-related modulators (*DTL*, *DTX3L*, *KCNMB1*, *NDRG1*, *GPX2*, *DERL3*, *MBTPS2*), after the patients with SKCM were stratified according to risk score, the somatic mutation pattern was also changed. Specifically, the possibility of variant was higher in the LRG (92.97%) than in the HRG (85.51%). Nevertheless, in the risk groups, the mutations were predominantly multiple hits and missense mutations, and the genes predominantly mutated were *TTN* and *MUC16*. (**Figures 8D, E**). Moreover, a total of 120 IC50 discrepant drugs were obtained in the risk group, and it was noteworthy that there were 18 markedly correlated discrepant drugs (|cor| ≥ 0.3) (**Table 4**). Visualization of the top 6 drugs with the highest correlation revealed that the IC_50_ values of all these drugs differed significantly between the high- and low-risk groups, suggesting that patients in the low-risk group may be more sensitive to these agents (**Figure 8F**). Overall, the differences in drug sensitivity between risk groups further highlighted the importance of risk-stratified treatment for SKCM patients, which might enhance therapeutic efficacy and reduce drug resistance.

**Table 4:** Correlation between 18 kinds of IC50 discrepancy drugs and risk score.

No.	Drug	Cor
1	Lapatinib_1558	-0.327
2	OSI-027_1594	-0.320
3	ERK_2440_1713	-0.314
4	ERK_6604_1714	-0.303
5	ZM447439_1050	0.307
6	Tozasertib_1096	0.320
7	BPD-00008900_1998	0.331
8	KU-55933_1030	0.433
9	Ribociclib_1632	0.433
10	AMG-319_2045	0.442
11	RO-3306_1052	0.481
12	BMS-754807_2171	0.500
13	AZD8055_1059	0.511
14	JQ1_2172	0.523
15	AZD6482_2169	0.529
16	Doramapimod_1042	0.540
17	NU7441_1038	0.584
18	SB216763_1025	0.622

### 3.8 Complex molecular regulatory network of prognostic genes

**Figure 9:**
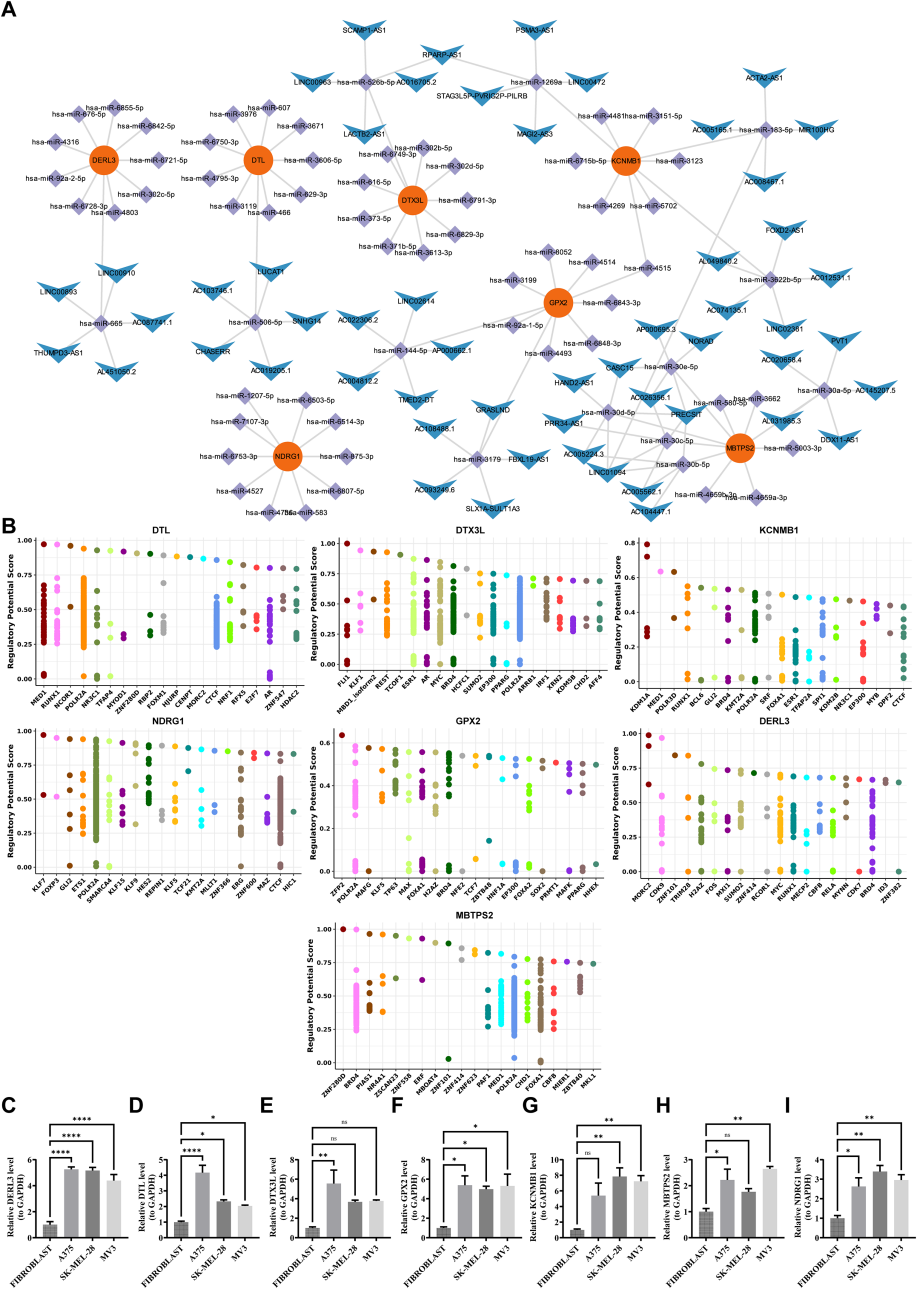
Regulatory networks of characteristic genes and PCR validation. **(A)** ceRNA network. Red nodes represent key genes, purple nodes represent miRNAs, and blue nodes represent lncRNAs. Connecting lines indicate regulatory relationships among them. **(B)** Transcription factor prediction. The predicted transcription factors targeting *DERL3*, *DTL*, *DTX3L*, *GPX2*, *KCNMB1*, *MBTPS2*, and *NDRG1* are shown. **(C-I)** Validation of prognostic genes by RT-qPCR. Expression levels of *DERL3*, *DTL*, *DTX3L*, *GPX2*, *KCNMB1*, *MBTPS2*, and *NDRG1* were experimentally verified.ns means not significant, *p means p < 0.05, **p means p < 0.01, **** means p < 0.0001.

A total of 278, 133, 4278, 133, 47, 285, 26, 191 and 149 miRNAs were identified to target *DTL*, *DTX3L*, *KCNMB1*, *NDRG1*, *GPX2*, *DERL3* and *MBTPS2*, respectively. Based on these results, 87 lncRNAs that target these miRNAs were obtained. *MBTPS2* had the most complex regulatory network and shared a common lncRNA with *KCNMB1* (LINC01094) (**Figure 9A**). Furthermore, the potential TFs of prognostic genes were further gained from the database. Specifically, the potential transcription factors for *DTL*, *DTX3L*, *KCNMB1*, *NDRG1*, *GPX2*, *DERL3* and *MBTPS2* were MED1, FLI1, KDM1A, KLF7, ZFP2, MORC2 and ZNF280D, respectively (**Figure 9B**). Briefly, molecular regulatory networks were mined, providing a basis for targeting and regulating prognostic genes.

### 3.9 Human melanoma cell lines express high levels of prognostic genes

Compared to fibroblasts, prognostic genes were significantly up-regulated in 3 human melanoma cell lines, especially *GPX2*, *DERL3*, *NDRG1*, and *DTL* (*p* < 0.05) (**Figures 9C–I**). In addition, RT-qPCR presented that the expression trends of prognostic genes and prognostic genes in immunohistochemistry were similar, further demonstrating the consistency of the analyzed results. In short, ISR-related prognostic genes had excellent diagnostic performance for SKCM samples, which provided new ideas for the diagnosis and targeted therapy of SKCM.

## 4 Discussion

SKCM is a highly invasive malignant cancer, and its prognosis is strongly correlated with stress adaptation within the tumor microenvironment (33). ISR, as a core mechanism for cells to cope with various stresses, plays a pivotal role in tumor progression and therapeutic resistance (34). Cancer cells harness the ISR to adapt to hostile microenvironments characterized by metabolic stress and hypoxia. As evidenced in: hepatocellular carcinoma, where eIF2α phosphorylation triggers aberrant ISR activation, driving tumor progression and conferring therapy resistance (35); lung cancer, wherein chronic stress stimuli activate ISR through the HIF1A-AS3/HIF-1α axis, promoting tumorigenesis via macrophage reprogramming (36). Concomitantly, novel ISR-targeting therapeutic strategies have been developed, exemplified by the biomimetic nanoplatform PPMC@CM deployed in melanoma. This system achieves concomitant suppression of immunosuppressive checkpoints and stress-adaptive signaling through co-delivery of: CRISPR/Cas9 for PD-L1 ablation, and MnO_2_ nanoparticles for hypoxia alleviation, thereby potentiating therapeutic efficacy against advanced melanoma (37). In this study, we identified seven prognostic genes, including *DTX3L*, *GPX2*, *KCNMB1*, *DERL3*, *NDRG1*, *DTL*, and *MBTPS2*, and constructed a novel prognostic model. Moreover, we investigated the correlation between these genes, the level of immune infiltration, and the efficacy of immunotherapy in SKCM. These results provide novel perspectives on the molecular mechanisms underlying the relationship between the ISR and SKCM, and contribute to the enhancement of SKCM prognosis and the identification of novel therapeutic approaches.

Among the prognostic genes identified, *GPX2* (Glutathione Peroxidase 2) is a crucial member of the glutathione peroxidase (GPX) family. Its primary function involves the elimination of hydrogen peroxide (H_2_O_2_) and lipid peroxides through the catalytic reduction of glutathione (GSH), thereby protecting cells from oxidative stress damage (38). In metabolically active melanoma cells, high levels of reactive oxygen species (ROS) constitute a significant feature (39), while the antioxidant enzyme GPX2 maintains intracellular redox homeostasis by clearing ROS, directly promoting the survival and proliferation of melanoma cells (40); In addition, considering that ROS is an important messenger for key signaling pathways such as MAPK, PI3K/AKT, and NF - κ B (41–43), GPX2 may indirectly affect these pathways by regulating ROS levels, thereby regulating the fate of tumor cells. In the tumor immune microenvironment, the role of GPX2 is equally crucial: on the one hand, it may affect immune cell function by regulating the ROS levels of tumor cells themselves. ROS can inhibit T cell activation, proliferation, and cytotoxicity (44), thereby helping tumors achieve immune escape. On the other hand, cytotoxic T lymphocytes (CTLs) require precise ROS regulation when delivering cytotoxic particles through immune synapses (45), so the expression of GPX2 in T cells or ROS levels in the tumor microenvironment may directly affect the killing efficiency of CTLs. GPX2 not only directly protects tumor cells from ROS invasion, but also weakens anti-tumor immune response by disrupting T cell functionality, jointly promoting the progression and immune escape of melanoma. This finding is consistent with our results, further indicates that low expression of *GPX2* may impair cellular capacity to scavenge reactive oxygen species (ROS), thereby resulting in the accumulation of oxidative stress and genomic damage, which ultimately facilitates tumor progression.

*DTL*, a homolog of E3 ubiquitin ligase, plays a crucial role in maintaining genomic stability. Research has indicated elevated expression of *DTL* in various cancer types and could operate as a HIF-1α-dependent effector within the hypoxia-triggered ISR axis, promoting tumor progression (46–48). Xuan’s research has found that *DTL* is significantly associated with poor prognosis in SKCM. Additionally, forced overexpression of *DTL* enhances the malignant biological activity of melanoma cells (49). Based on these findings, this study further explored the molecular mechanisms underlying *DTL*’s role in promoting the development and progression of cutaneous melanoma. It was discovered that high *DTL* expression may drive tumor progression by accelerating cell cycle progression and inhibiting apoptosis caused by defects in DNA repair. Similar to DTL, DTX3L is also an E3 ubiquitin ligase widely involved in cell cycle regulation and DNA damage repair processes, playing an important role in the occurrence and development of various tumors (50, 51). In recent years, research has found that DTL plays a key role in the evolution of melanoma. Its pro cancer mechanism mainly includes two aspects: on the one hand, DTL promotes the malignant progression of melanoma by reshaping the glucose metabolism process of tumor cells (46); On the other hand, it drives the proliferation and migration of melanoma cells via the ERK/E2F1/BUB1 signaling axis (49). In addition, clinical sample analysis showed that high levels of DTL expression in circulating cell-free RNA (cfRNA) were closely associated with significantly shortened survival in patients (52), further supporting its potential as a prognostic biomarker. In summary, DTL and DTX3L have multiple pro cancer functions in the development of CM, not only participating in metabolic recombination and signal transduction regulation, but also having important clinical translational value, suggesting that they can serve as potential diagnostic markers and therapeutic targets.

*KCNMB1* (calcium-activated potassium channel subfamily M regulatory beta subunit 1), as a pivotal regulatory subunit of BK channels, primarily controls the vascular smooth muscle and maintains intracellular calcium homeostasis by regulating intracellular calcium ion levels. It effectively prevents calcium overload in both the endoplasmic reticulum (ER) and mitochondria, which are key triggers of the ISR (53–55).The BK channel and its regulatory subunit KCNMB1 play multiple key roles in melanoma. Firstly, KCNMB1 directly regulates the proliferation and survival of melanoma cells by affecting the cell cycle progression and apoptosis signaling pathway (56); Secondly, based on its important role in regulating vascular function in vascular smooth muscle (57), KCNMB1 is likely to provide support for the growth of melanoma by regulating tumor related blood vessels; In addition, considering that the progression of melanoma is highly dependent on its immune microenvironment (58), the widespread presence of BK channels in immune cells suggests that KCNMB1 may participate in shaping a favorable microenvironment for tumor immune escape by regulating immune cell function. KCNMB1 may promote the progression of melanoma by synergistically regulating the autonomous function of tumor cells, tumor blood vessels, and immune microenvironment.

*NDRG1*, an N-myc downstream-regulated gene, encodes a multifunctional protein primarily involved in organogenesis and cell differentiation (59, 60). Previously, Gao Y et al. found that *NDRG1* is downregulated in SKCM, negatively correlated with the HIF-1α signaling pathway, and involved in the ferroptosis process induced by ultraviolet exposure (61). The loss of *NDRG1* expression may accelerate tumor progression by promoting angiogenesis. In this study, we observed that *NDRG1* exhibits an independent association with a more favorable prognosis in SKCM, suggesting its potential role as a tumor suppressor gene. However, unlike previous findings, our qPCR results revealed high expression levels of *NDRG1* in skin melanoma tissues. This disparity may be ascribed to a variety of factors such as sample differences, tissue sampling locations, and the multifaceted regulation of genes. Further validation at the protein and metabolic levels is warranted. Additionally, as a key component of endoplasmic reticulum-associated degradation (ERAD), *DERL3* directly binds to activating transcription factor 4 (*ATF4)* to enhance ERAD capacity for clearing misfolded proteins, thereby suppressing excessive ISR activation (62). Meanwhile, *MBTPS2* participates directly in ISR regulation through the cleavage of activating transcription factor 6 (*ATF6*) (63). Our findings demonstrate that both genes exhibit prognostic trends in SKCM similar to that of *NDRG1*.

To understand the related functions of prognostic genes, we conducted the single-gene GSEA enrichment analysis. The results showed that the genes *DTX3L*, *KCNMB1*, and *DERL3* were strongly correlated with immune - associated pathways, including chemokine signaling and cytokine-cytokine receptor interactions. Additionally, the genes *DTL*, *MBTPS2*, and *NDRG1* were found to be strongly associated with cell cycle and DNA replication pathways. Research has indicated that chemokines and cytokines such as CCL4, CCL3, IFN-γ, and interleukins are expressed at notably higher levels in SKCM patients than in healthy individuals. Furthermore, it was observed that CCL3 levels gradually increase with the progression of SKCM, providing compelling evidence for the involvement of chemokine and cytokine signaling pathways in promoting the development of SKCM (64). In tumor cells, the cGAS-STING pathway mediates the production of interferons and proinflammatory factors, thereby enhancing the recruitment and activation of immunocytes in the tumor-microenvironment. While *DTX3L* inhibits antitumor immune responses by mediating the ubiquitination and degradation of cGAS protein (65). This implies that targeting the *DTX3L*-cGAS axis holds the potential to enhance the prognosis of patients with SKCM by augmenting anti-tumor immunity. Meanwhile, *DTL* was significantly enriched in both cell cycle and DNA replication pathways. Its high expression may promote tumor cell proliferation by maintaining the stability of DNA replication forks and accelerating the S-phase process (49). Additionally, through PPI analysis, this study found a strong interaction between *DTL* and RAD51 protein, indicating its potential involvement in homologous recombination repair and its impact on genomic stability, which may drive treatment resistance in SCKM (66). These findings align with the significantly reduced survival rate discovered in the high-risk group in this study, highlighting the potential of *DTL* as a therapeutic target. It is noteworthy that low expression of *GPX2* leads to the inhibition of the glutathione metabolism pathway, weakening antioxidant defense, inducing the accumulation of reactive oxygen species (ROS), and subsequently activating the NF-κB pathway. This promotes the release of proinflammatory factors and the formation of an immunosuppressive microenvironment.

Skin melanoma is widely recognized as one of the most immunogenic cancers, and its unique immune micro-environment contributes to poor immunotherapy responses as well as the tumor’s strong invasiveness and proliferative capacity (67). Through immune infiltration analysis, we identified 38 differentially infiltrating immune cells among risk groups, indicating that alterations in the immune microenvironment might be modulated by prognostic genes. For instance, *GPX2*, through ROS accumulation, activates the TGF-β signaling pathway (68) and promotes the infiltration of M2 macrophages and Treg cells, building an immunosuppressive network. Additionally, *DER3L* and *KCNMB1* are closely related to the infiltration levels of plasma cells and Th1 cells respectively. To explore immunotherapy response, ESTIMATE analysis was conducted, revealing that the low-risk group corresponded to higher stromal and ESTIMATE scores. Generally, a higher stromal score is associated with a poorer immunotherapy response (69). Similar results were observed in the TIDE analysis, where the low-risk group had higher overall TIDE scores and immune dysfunction scores. This suggests that tumors in this group may have stronger immune evasion potential and possible T-cell dysfunction within the tumor micro-environment, indicating a lower benefit from immunotherapy for the low-risk group. However, these are not the only predictive factors, as other elements such as the complex micro-environment of the tumor itself and tumor-specific immune responses also play crucial roles, necessitating further experimental validation. Additionally, through differential analysis of immune checkpoints, we found significant differences across almost all checkpoints, most of them exhibited relatively higher levels within the low-risk group. These findings emphasize the significance of patient stratification and personalized treatment.

In recent years, the potential value of dietary supplements in chronic disease management has been increasingly recognized. Research has shown that specific nutrients may play an important role in maintaining tissue homeostasis by regulating gene expression and enhancing tissue stress adaptation. For example, studies have found that lutein pigments can improve eye health by regulating stress response pathways in the retina (70). This mechanism suggests that dietary supplements may also affect the microenvironmental adaptation and disease progression of SKCM by regulating ISR related genes. The ISR related prognostic genes identified in this study, including GPX2 involved in oxidative stress regulation, DTL associated with DNA replication repair, and DERL3 regulating endoplasmic reticulum stress, are all key molecules in cellular stress response. There is evidence to suggest that dietary components such as carotenoids may enhance the antioxidant defense system (71), affect the expression of genes such as GPX2, thereby reducing oxidative damage and inhibiting tumor development. In addition, micronutrients such as vitamin D have been shown to regulate the stress adaptation ability and treatment sensitivity of melanoma cells (72–74). These findings provide mechanistic support for the feasibility of nutritional intervention as an adjuvant strategy for melanoma. However, the use of dietary supplements in tumors still needs to be carefully evaluated. Certain components may have a pro tumorigenic effect in high stress microenvironments, such as high-dose antioxidants that may even promote tumor metastasis in specific situations (75). Therefore, it is necessary to combine metabolomics and single-cell technology in future research to systematically evaluate the effects of different nutrients on the ISR pathway and tumor immune microenvironment.

Based on the ISR related risk stratification model constructed in this study, we divided SKCM patients into HRG and LRG, providing a key tool for achieving precise typing in the era of immunotherapy. The core clinical value of this model lies in its ability to go beyond traditional pathological parameters and introduce ISR as a new biological dimension. Although the predictive performance of the model is at a moderate level (C-index=0.71) and has not been directly compared with mature systems such as AJCC staging or DecisionDx Melanoma (76, 77), its unique advantage lies in achieving a leap from prognosis assessment to treatment guidance. The model can not only identify subgroups of patients with different prognoses within the same AJCC stage, but also reveal the essential differences in the immune microenvironment between two groups of tumors. For HRG patients with poor prognosis, their relatively low TIDE scores suggest that the tumor microenvironment may be more easily reversed by immune checkpoint inhibitors and should be considered as a priority candidate for immunotherapy. For LRG patients with good prognosis, their higher immune dysfunction scores indicate that they may not respond well to monotherapy immunotherapy, and clinical attention should be paid to drug resistance and explore combination therapies. In addition, the inter group drug sensitivity differences suggested by the model provide new directions for targeted therapy selection. In summary, as a “prognostic predictive integrated” biomarker, this model provides a direct and transferable decision-making basis for individualized stratified treatment of melanoma. The prognostic classification and treatment guidance potential demonstrated by this model provide valuable directions for further exploration in achieving more valuable patient stratification in the precision treatment system of melanoma in the future.

To summarize, our research systematically identified a novel prognostic gene signature associated with the integrated stress response (ISR) in cutaneous melanoma, demonstrating its prospective utility as a prognostic instrument for patient categorization and therapeutic decision-making. Through a combination of bio-informatics analysis and experimental validation, we untangled the functional relevance of key genes (e.g., *DTL* and *DERL3*) in melanoma progression and stress adaptation. However, there are several limitations to this study that need to be addressed: Firstly, this study has some limitations. Retrospective data analysis may introduce selection bias, and there may be some imbalance in sample grouping, which may affect the stability and statistical effectiveness of model evaluation. Secondly, the universality of the model needs further verification, especially in the specific subtypes of melanoma and non-Caucasian populations, where its performance is still unclear, and the constructed network regulation has not undergone functional experiments. In addition, there are inconsistencies between the expression results of some genes and previous studies, such as immunohistochemistry showing high expression of NDRG1 in SKCM tissues, while some literature reports low expression. This difference deserves further exploration. In terms of experimental verification, although RT qPCR revealed gene expression trends, it is difficult to fully confirm its specificity for melanoma using fibroblasts as a control; Meanwhile, the number of biological replicates is limited, and there is a lack of primary tissue evidence, systematic validation at the protein level, and functional experiments to clarify their causal relationships. Finally, although database based drug sensitivity prediction suggests inter group differences, its results are derived from *in vitro* models and cannot fully reflect the true drug response in patients. In response to the above issues, future research should focus on expanding the sample size, paying special attention to including high-risk patients, and conducting multicenter prospective cohort studies to verify the robustness and generalization ability of the model. In terms of experimental design, normal human melanocytes were used as controls, and biological replicates were added to rigorously verify the specificity of gene expression; And using gene editing techniques such as CRISPR/Cas9, we will delve into the functions and mechanisms of key genes in *in vitro* and *in vivo* models. At the same time, the expression of prognostic genes should be systematically validated at the protein level, and their cell specific distribution in the tumor microenvironment should be analyzed using techniques such as single-cell transcriptomics. In addition, the efficacy prediction ability of the model should be evaluated in large immunotherapy cohorts, and its combined application value with existing biomarkers should be explored. For drug prediction results, systematic validation should be conducted through *in vitro* and *in vivo* experiments, and retrospective and prospective analysis should be carried out in combination with clinical medication data to enhance their clinical translational potential. Ultimately, by developing a combined strategy of targeted ISR pathways and immunotherapy, it is expected to provide new treatment options for SKCM patients, promote the clinical translation of prognostic models, and assist in the precise diagnosis and treatment of melanoma.

## Data Availability

The original contributions presented in the study are included in the article/**Supplementary Material**. Further inquiries can be directed to the corresponding authors.
