# Supplementary figures and images for: Identification and validation of prognostic genes and prognostic models associated with cutaneous melanoma and integrative stress response
Source: Front Immunol. 2025 Dec 2;16:1689103. doi: 10.3389/fimmu.2025.1689103 (PMC12705592; doi:10.3389/fimmu.2025.1689103)

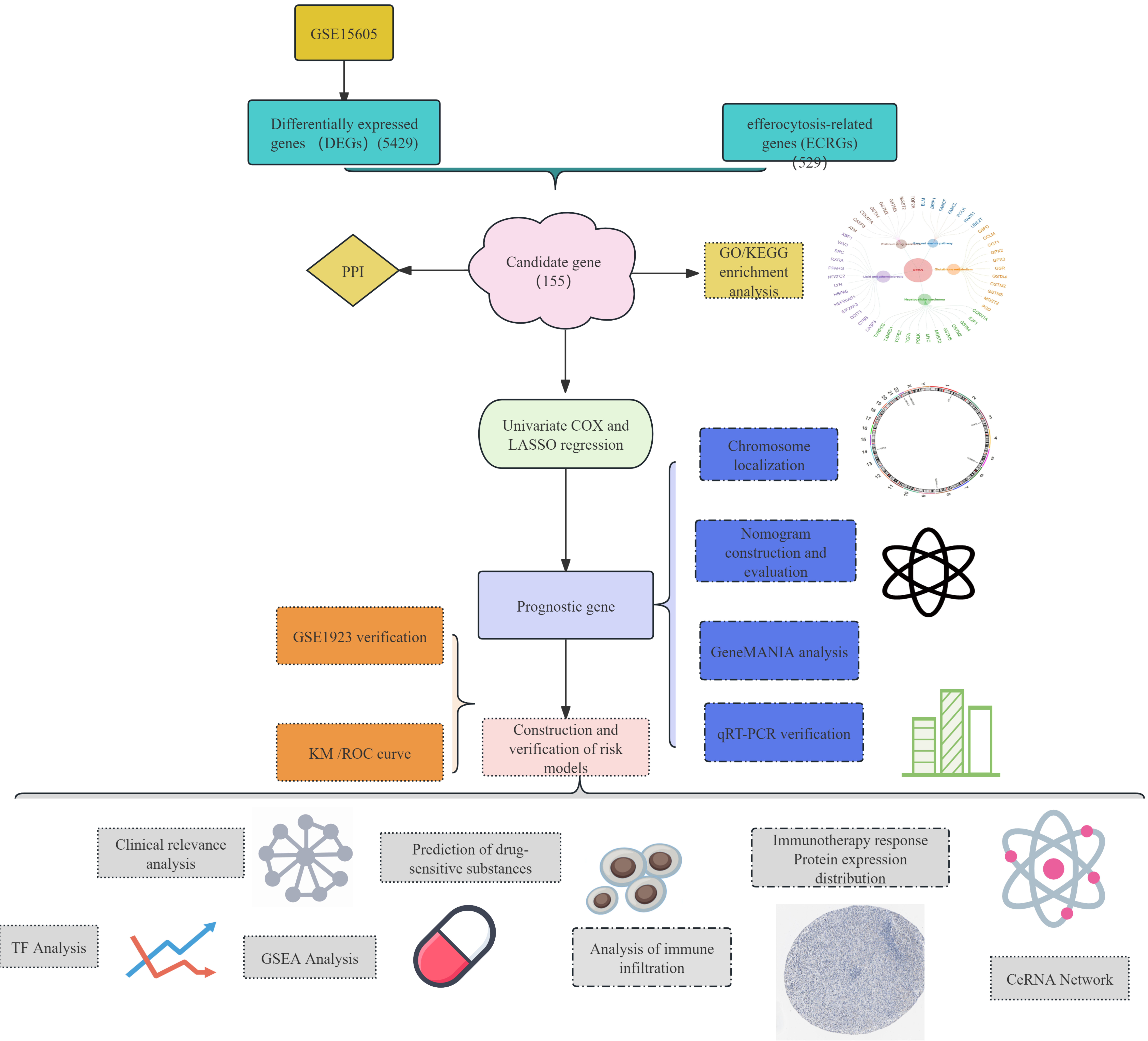

Supplement: Supplementary file 1 [file DataSheet1.pdf]

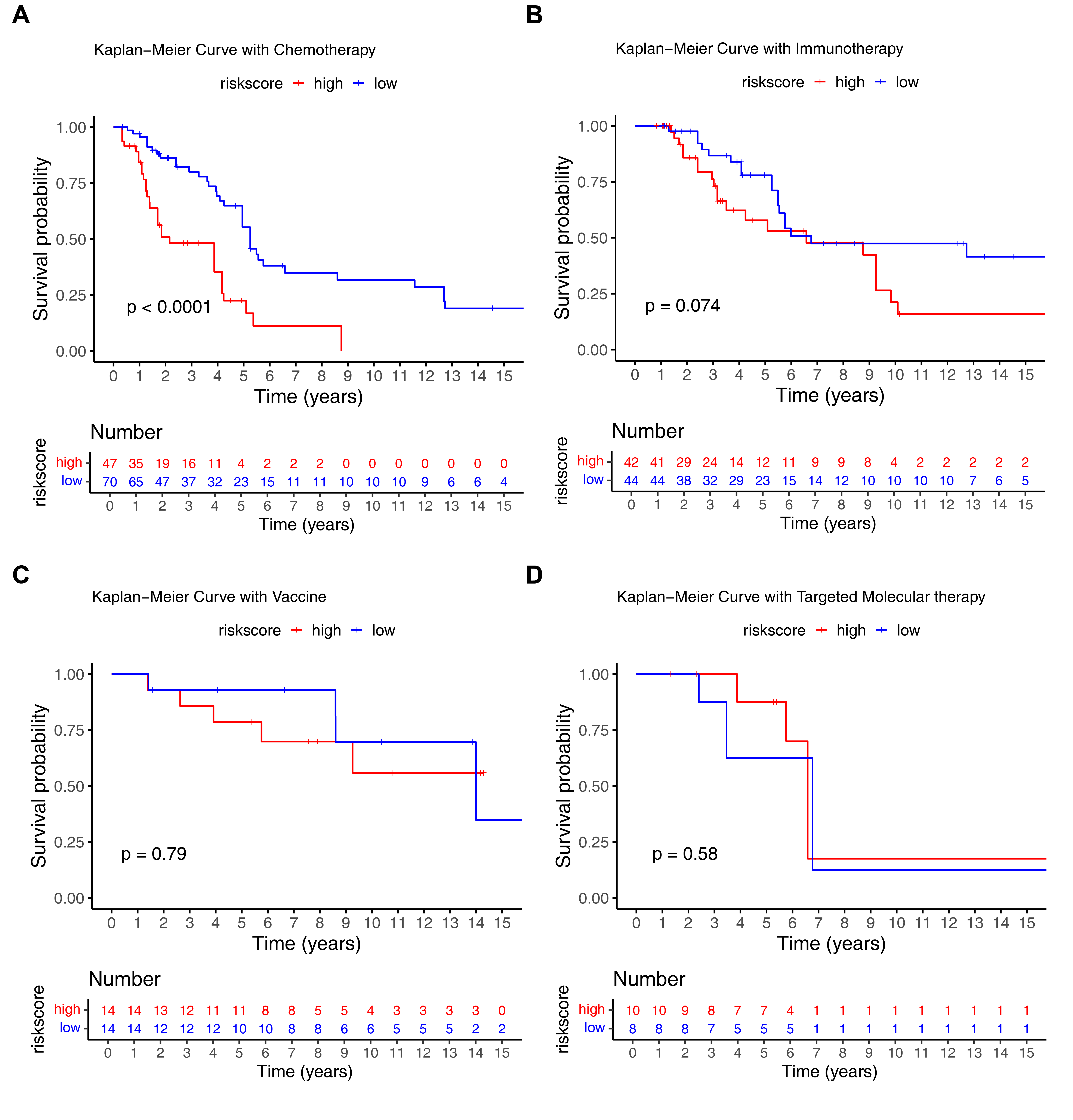

Supplement: Supplementary file 2 [file Image1.tif]
