# Supplementary material for: Identification and validation of prognostic genes and prognostic models associated with cutaneous melanoma and integrative stress response
Source: Front Immunol. 2025 Dec 2;16:1689103. doi: 10.3389/fimmu.2025.1689103 (PMC12705592; doi:10.3389/fimmu.2025.1689103)
Supplement: Supplementary file 15 [file Table13.docx]

**Supplementary Fig.1** The predictive efficacy of the ISR risk model for different treatment regimens. **(A)** Survival curves for each group of high and low risk based on chemotherapy. **(B)** Survival curves for each group of high and low risk based on immunotherapy. **(C)** Survival curves of each group with high and low risks based on vaccine treatment. **(D)** Survival curves of each group with high and low risks based on targeted molecular treatment.
